# Epigenetics: Its Understanding Is Crucial to a Sustainable Healthcare System

**DOI:** 10.3390/healthcare3020194

**Published:** 2015-04-01

**Authors:** Michelle Thunders

**Affiliations:** College of Health, Massey University, Wellington 6140, New Zealand; E-Mail: m.thunders@massey.ac.nz; Tel.: +64-4-979-3461

**Keywords:** epigenetics, health, disease

## Abstract

Understanding the molecular impact of lifestyle factors has never been so important; a period in time where there are so many adults above retirement age has been previously unknown. As a species, our life expectancy is increasing yet the period of our lives where we enjoy good health is not expanding proportionately. Over the next 50 years we will need to almost double the percentage of GDP spent on health care, largely due to the increasing incidence of obesity related chronic diseases. A greater understanding and implementation of an integrated approach to health is required. Research exploring the impact of nutritional and exercise intervention on the epigenetically flexible genome is up front in terms of addressing healthy aging. Alongside this, we need a greater understanding of the interaction with our immune and nervous systems in preserving and maintaining health and cognition.

## 1. Introduction

We are products of our ancestry, our genetic inheritance and also our behaviour and environment; these are intimately intertwined and the molecular understanding of how the two interplay is now starting to be explained by the field of epigenetics. Epigenetics is the study of changes that do not involve alterations to the genetic code, but are often still passed down to future generations. As its name suggests, the epigenome exerts its effect of gene control through a variety of mechanisms that essentially physically block or reveal areas of the genome concerned with gene regulation. It is through the epigenome that external factors affect our genes. Epigenetics looks at how our predetermined genotype interacts with our environment or vice versa to produce our through-life phenotype that has an element of plasticity to it. Your genome and genotype remain unchanged throughout your life course where as your physical phenotype and your epigenome (the product of your genome interacting with your environment) change as you age. Some of the changes in the epigenome are transient and act as a flexible homeostatic mechanism that only affects the individual; others appear to be heritable; with mother to offspring transmission of epigenomic control being observed particularly with regards to obesity, birth weight and health of successive generations [1].

**Figure 1:**
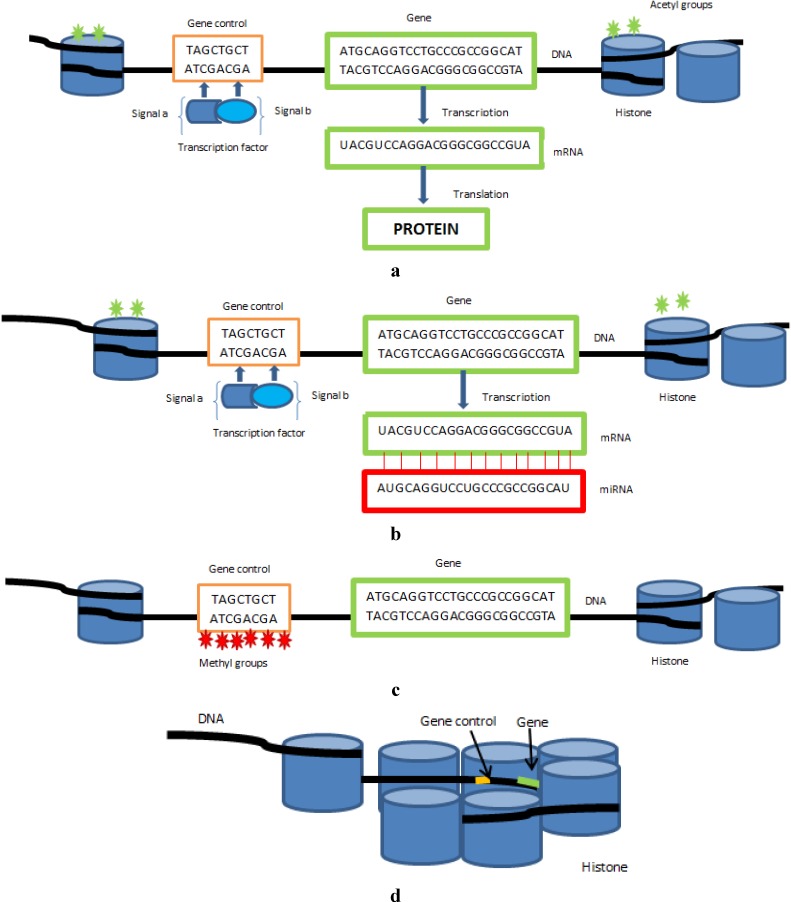
(**a**) illustrates a gene being actively transcribed to produce a protein; (**b**) illustrates how microRNAs affect gene control through binding to the gene’s transcribed mRNA preventing it from being translated into a protein; (**c**) illustrates how methyl groups bind to areas preceding a gene responsible for regulating gene control and prevent transcription factors etc. binding to the control region thus repressing gene expression; (**d**) illustrates how histone deactetylation and chromatin remodeling affects gene expression, condensed chromatin where DNA is tightly coiled around histones makes genes inaccessible to regulatory proteins and thus results in gene repression.

Epigenetic modifications and miRNAs work through a complex and intricate molecular communication network to enhance or suppress gene expression. The mechanisms that mediate epigenetic regulation are principally DNA methylation, the post-translational modifications of histones, chromatin re-modelling and the regulation by non-coding RNAs (Figure 1). They are intimately related to cell differentiation and developmental plasticity and relay environmental influences to the cell nucleus, thus bridging the gap between lifestyle and the genome. The primary role for epigenetic control of genes is to silence transcription of genes so they are not expressed where they are not needed. This is vital in embryonic development and errors in epigenetic control mechanisms result in embryonic lethality [2]. Epigenetic control has another function; it orchestrates the body’s response to environmental stimuli. There has been much interest whereby such changes to genome control are passed on through generations, as in the Dutch famine study [3]. The underlying premise is that extreme circumstances experienced by the mother in the early stages of pregnancy effectively prime the foetus for a harsh environment and as such the foetus makes adaptive metabolic changes in utero through epigenetic control of genes involved in metabolic pathways. What determines the carry over and length of duration of such trans-generational epigenetic imprints, and the timeline for health consequences to manifest at the population level, is yet unknown but could have profound implications for the health advice we give. This could be particularly pertinent with reference to the health impact for successive generations and become a major consideration in healthcare budget forecasting. Being able to foresee and prevent or reduce potential future healthcare burdens from arising could help minimize “bow wave” funding crises and kneejerk reactions in terms of healthcare advice and spending. There are so many questions in this area yet to be answered, for example, are trans-generational epigenetic imprints a mechanism whereby recently migrated populations initially mal-adapt metabolically? If so, this could be an important area of research to focus on to improve health equity within a population where efforts are targeted on reversing the metabolic mal-adaptation through manipulating the epigenome’s malleability and role in molecular homeostasis. As well as a role in trans-generational control of gene expression, epigenetics also has a role in the much more rapid and dynamic adaptive response to environmental stimuli. Implications for this are that future therapeutics directed at optimising metabolic health could be focused on lifestyle changes that bring about changes in metabolism through flexible epigenetic mechanisms.

**Figure 2:**
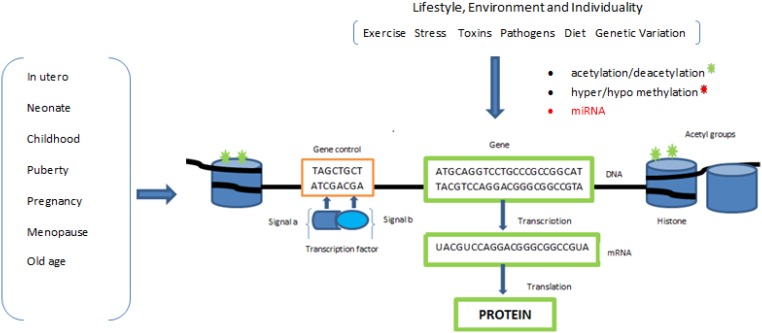
Illustrates how transient epigenetic effects contribute to regulating gene function. Lifestyle, environment and individuality all have an effect on gene regulation through the epigenetic mechanisms described; this type of molecular homeostatic control is likely to vary at different periods during our lifetime, particularly in periods of rapid growth and our individual epigenome changes as a function of age.

A key function of epigenetic regulation is to ensure gene expression is specific to cell and tissue type, if this mechanism of cell differentiation control is disrupted then this can have serious consequences for the ensuing phenotype and has been highlighted as a contributing factor to many diseases. However, there is now evidence that the epigenome is susceptible to a range of environmental cues such as variations in diet, maternal behaviour or stress which are particularly evident during specific developmental periods [4]. The environmental sensitivity of the epigenome has been suggested to reflect an adaptive mechanism, by which the organism can adjust its metabolism and homeostatic systems to suit the environment, in order to aid survival or reproductive success in adulthood (Figure 2). Inappropriate adaptation has been linked to the development of a range of chronic diseases in later life and has been suggested to account for at least some of the rapid increases in the rates of obesity, Type 2 Diabetes Mellitus (T2DM) and cardiovascular disease recently observed in both developed and developing countries.

Exercise is widely acknowledged to have an immense impact on health, general well-being and life span. Lack of physical activity has also been linked with the progression of many chronic diseases. Research suggests that epigenetic changes occur in response to acute resistance and aerobic exercise in a range of tissues including skeletal, blood, adipose and brain and with 6 months of aerobic exercise showing alterations of whole genome methylation in skeletal muscle and adipose tissue directly influencing adipogenesis [5]. The mechanisms through which exercise is beneficial to our health have been largely classified as either anti-inflammatory or immune-modulatory. The way in which exercise brings about these changes, both acute and longer term, is through the interaction with the homeostatic control of our genome and our gene expression, largely through epigenetic control. The process of exercise enhances and in some cases “reawakens” cellular and molecular processes that cause tissues to change and adapt and along with these changes comes the associated beneficial effects of exercise.

## 2. Epigenetics, Inflammation, Nutrition and Metabolism

A lot of research effort has been spent looking at the association between inappropriate inflammation and chronic metabolic disease, with the cellular profile of adipose tissue altered such that it is in a state of hyper inflammation, a process believed to contribute to insulin resistance [6]. Increase in fat mass in obese individuals has a major role in low level chronic inflammation, insulin resistance and storage of lipids in the liver. Why this occurs is assumed to be an adaptive response to calorific overload with adiponectin having a central role in trying to reduce the hyper-inflammatory response. In obese patients adiponectin levels are low, it decreases as liver fat content increases, and as such these individuals are not able to get a control on the increased inflammation measured through levels of IL-6, TNF-alpha, IL-2, and IFN-gamma [7]. Recent work looking at the effect on gene expression of acute exercise has postulated that the immune reaction observed after intense exercise is of a protective anti-inflammatory nature [8]. Even very short bouts of exercise, 10 × 2 min bouts of cycle exercise at an average of 82% maximum oxygen consumption interspersed with 1 minute rest, have been demonstrated to have an impact on gene expression of genes involved in vascular health. It is not known yet how long these changes last but provides important evidence emphasising the importance of exercise to achieve and maintain cardiac health and also possibly rethinking our attitude to exercise recommendations [9].

It remains important to elucidate the anti-inflammatory mechanisms through which exercise prevents the development of chronic disease, the precise nature of control on the inflammatory profile of adipose tissue and maintenance of insulin sensitivity is crucial in maintaining and increasing the healthy living portion of our lives and vital in developing a weight loss independent mechanism for addressing the increasing burden of insulin resistance. There is lot of research currently focusing on the microbiome and the role of intestinal flora in influencing body weight. The hypothesis being that a shift in gut flora can readjust the inflammatory balance and contribute to controlling the hyper inflammation and restore insulin resistance without the need for drastic weight loss [10]. This in part is evidenced by patients who have undergone gastric bypass surgery and have had gut microbiota restored to a composition similar to patients who have undergone prebiotic treatment or weight loss following calorie restriction [11]. This area needs much more research but is intriguing in the linkage between microbiology, chronic disease and inflammation and provides an additional jigsaw piece in the complex puzzle of chronic metabolic disease.

Not surprisingly genes involved in homeostatic process and metabolism are the sites most likely to display the largest epigenetic variation [12]. Recent studies [13] have suggested that mitochondrial dysregulation is a major common factor in T2DM and obesity, a decreased functionality of mitochondria arising as a consequence of an overload in nutrition. This stress to the mitochondria in turn is associated with insulin resistance and disrupted lipid metabolism. This appears to be reversible and studies suggest that through restricting calorie intake or through adapting to a more physically active lifestyle, the function and integrity of mitochondria can be significantly improved and thus protect against further metabolic disease [14]. This relationship between lifestyle factors and functionality of the mitochondria suggest there is a significant epigenetic component to the regulation of their function. PGC1 alpha is known as an important regulator of mitochondrial biogenesis and a key regulator of energy metabolism. As such it is sensitive to environmental factors as well as varying with gender, age, birth weight, and aerobic capacity. The activity of the protein can be regulated by methylation of the gene promoter region. Increased methylation in this region being found in multiple tissues of patients with T2DM compared with normal glucose tolerant subjects. In the T2DM patients this results in reduced gene expression and reduced number of mitochondria, which in turn results in impaired insulin secretion from pancreatic islets and reduced insulin sensitivity in the liver. Exercise intervention has been found to reduce promoter methylation of a number of genes key in the regulation of metabolism including PGC1-alpha in human and rodent skeletal muscle leading to increased up-regulation of the gene relative the amount of exercise performed [15].

## 3. Epigenetics Exercise and Lifestyle

Increasing evidence suggests that exercise is a crucial orchestrator of the transcriptome through its epigenetic regulation of expression of a plethora genes involved in transcription factors, co-factors and activators of metabolism, mitochondrial biogenesis and function [16]. Skeletal muscle adapts to a single bout of acute exercise, the mechanics by which skeletal muscle responds involves a multitude of molecular signaling cascades involved in metabolism, protection against oxidative stress, immune-regulatory and hormone signaling to name but a few. The type, intensity and duration of the exercise determine the specific and relative contribution of each intracellular process [17]. For example, with exercise that has a high power output, like lifting weights, there will be higher calcium efflux and greater downstream calcium-dependent protein kinases, compared to endurance exercise. The action of the kinases is then to phosphorylate histone deacetylases which consequently relaxes the chromatin structure facilitating increased gene expression. Intracellular calcium increase is a necessary contributory process to DNA de-methylation.

HDAC4 is a histone deacetylase known to repress GLUT4 transcription in adipocytes, in skeletal muscle during exercise suggesting that removal of transcriptional repression could be a mechanism whereby exercise adaptation occurs. HDAC inhibitors have been suggested in treatment of obesity and T2DM [5]. In human and mouse models, methylation of PGC-1alpha, TFAM, MEF2A, CS and PDK4 promoters was decreased after a single acute exercise episode [18]. Exercise intervention can also bring about chronic changes to DNA methylation status; with muscle biopsies taken 48 hours after a 6 month intervention found to be differentially methylated in genes involved in a diverse range of metabolic pathways [5]. The majority of changes were to hypomethylation, suggesting that the hypomethylation was at least in part retained, but for how long is uncertain. This conversely proposes a model for chronic disease state in that if exposed to chronic conditions then hypermethylation status could also be retained, this is supported by a study demonstrating that physical inactivity is as detrimental on a molecular level as activity is beneficial, with as little as 9 days bed rest enough to increase insulin resistance and methylation of promoters in PGC-1alpha. These changes were shown to be partly reversed by a 4 week retrain showing the importance of physical activity in re-booting epigenetic control of genes key to metabolism [19]. Adaptation conferred by exercise involves epigenetic modifications; molecular training needs to take place as well as physical. Exercise responsive gene expression and the manipulation of the plasticity of our epigenome to respond to a changing environment will be the future for interventional health programs.

Weight loss, calorie restriction and dietary intervention have all been demonstrated as mechanisms that can improve mitochondrial biogenesis highlighting the importance of lifestyle interventions and their impact on our molecular health. The primary source for DNA methylation of our genome comes from our diet, specifically from dietary methyl donors hence the importance of nutrition in both the establishing and maintaining of methylation patterns [20]. Experiments in mice suggest that with the appropriate amount of exercise the calorific origin of energy is less important, mice fed high fat diets but still undertook exercise training were protected against the harmful changes in inflammatory profile seen in adipose tissue associated with obesity; possibly in part through the exercise preventing the expansion of the adipose tissue [21]. Acute exposure of human primary muscle cells to free fatty acids or inflammatory cytokines increases promoter methylation of genes involved in mitochondrial function [22], but what is more interesting, and crucial for future health care planning, is that in rodent studies the methylation levels were normalised once a normal diet was restored, suggesting that DNA methylation is reversible. Whilst our diet and physical activity are likely to have a big impact on the contribution of epigenetic flexibility there are many other environmental factors that are likely to contribute too. Methylation patterns have been shown to exhibit circadian rhythm, potentially due to the variation in homocysteine levels throughout the day [23]. The effect of disruption of the sleep-wake cycle also has been demonstrated to alter epigenetic gene control, potentially through the epigenetic re‑programming of circadian genes. A study in Italy on shift workers found the most significant results were relating to the position of seniority of the workers and their methylation status; potentially suggesting the role of stress in epigenetic control too. The disruption of epigenetic control in shift workers potentially could be an explanation for the increased health vulnerability of this population, particularly in terms of increased risk for cancer [24].

## 4. Epigenetics and Aging

Studies on a range of species including humans have shown that global genomic methylation decreases as we age [25]. Alongside this there are a number of specific gene sites where the promoter region becomes increasingly methylated with age including oestrogen receptor, IGF-2, and putative tumour suppressor genes. Longitudinal studies investigating the change in methylation with age have shown that there is familial aggregation in terms of patterns of increased and decreased methylation suggesting that the genotype too plays a role in determining to what extent our epigenome is flexible with our environment. Healthy aging is an increasingly topical issue in health sector funding; recent research has suggested that epigenetic mechanisms also play a role in determining the onset of age-associated diseases and lifespan potential. How we accumulate these age-related changes to our epigenome is yet to be fully elucidated, popular thought is that both stochastic and environmental factors are likely to play a role. Stochastic factors relating to the non-environmental random changes, likely due to increasing ineffectiveness of the genome and epigenome surveillance and maintenance proteins and enzymes. Nutrition, stress, exercise, pollution and endocrine disruptors are all environmental factors with potential to affect the epigenome. There has been much work supporting the importance of the early life environment in terms of the epigenomic health and future disease risk. These include studies on the impact of maternal nutrition on lifelong health and aging, even to the point where analysing epigenetic marks on cord blood can facilitate predicting childhood health [26]. What is really interesting is that the plasticity of our epigenome persists throughout our lives; studies in rats showing methyl deficient diets given to adult rats can produce hypomethylation of proto-oncogenes after just four weeks, and persisted 3 weeks after a normal diet is resumed [27]. This is likely to be more vulnerable at periods of rapid growth such as childhood, puberty and pregnancy. A study in mice showing folic acid supplementation had an effect at increasing methylation at age 18 months but no effects at age 4 months [28]. Macronutrients in adult rats showed enriching for fish oil in the diet for 9 weeks led to increase methylation of specific genes and could be reversed through a 4 week standard diet. Calorie restriction also has been reported to impact on epigenetic control, in species with a short life span restricting calories correlates with increasing longevity and in higher mammals is associated with the delay of age-related diseases. The mechanism is uncertain but potentially through either affecting or reversing methylation status [29]. There is increasing evidence to suggest that our epigenome is dynamic and changes in response to our environment throughout our lives, with evidence suggesting even fully differentiated somatic cells such as skeletal and muscle cells are able to respond to diverse environmental stimuli [30], the potential adaptive capacity of skeletal muscle is of particular interest to physical health with exercise intervention and innovative exercise based therapies used to enhance the health benefits of exercise on our epigenomic health and performance.

The point here is that rather than fretting on an approach that is focused on our differences maybe the key for future healthcare success is for us to understand the different approaches that can be employed to achieve a similar molecular metabolic endpoint. By focusing on just one of the multitude of contributing factors to health in isolation we are unlikely to ever produce successful population health results given the complexity of our homeostatic control over gene expression. If we continue to pursue and fund healthcare from individual discipline perspectives we are very unlikely to achieve success in enabling healthy populations. Maybe we should move towards diagnoses that are influenced by molecular epigenomic health. To achieve good health requires an integrated approach understanding how our environment, genes, individual physiology and actions interact and affect our metabolism positively or negatively through epigenetic control. Understanding the dynamic and long-lasting effects of exercise on epigenetic regulation of metabolism is likely to be crucial to integrating and addressing the multitude of factors that contribute to health. Epigenetic marks can also be transmitted to subsequent generations so it is possible that the effects of transient environmental exposure could be transferred to subsequent non-exposed generations, potentially having a cumulative effect in terms of change to gene control. Thus, there is the potential for children to experience the effects of their parents’ exposures to toxins, dietary excesses or insufficiencies and stressors. This could have huge implications in terms of assessing health risk. Physical exercise, likewise, may affect epigenetic modification and gene expression of not only the individual but also their progeny.

## 5. Conclusions

Physical exercise is likely to be the primary tool used to manipulate epigenomic metabolic re-booting. This field is exciting, and very much in the discovery phase, a more comprehensive analysis is needed to investigate the specific epigenetic responses to varying forms of physical activity at different intensities, frequencies and durations. The field of exercise epigenomics should expand rapidly as it can demonstrate how and why exercise benefits health and how we can manipulate our environment to optimise both our health and our performance. Our epigenome responds to the conditions it is set, getting healthy simply involves ‘rebooting’ our epigenome either through hard work or potentially in the future through development of pharmaceutical, nutriceuticals or even light activated implants [31] with the capability of targeting widespread metabolic gene expression. If we are to address the increasing burden to national healthcare systems from chronic obesity related diseases we need a much deeper understanding of the exquisite interplay between our genome, lifestyle and epigenome. If we are to shift the focus of healthcare funding towards healthy aging then it is imperative that more research is carried out to further our knowledge on the impact of nutritional and exercise intervention on the epigenetic control of our genome.
